# Medical Students’ Personalities: A Critical Factor for Doctor-Patient Communication

**DOI:** 10.3390/ijerph18179201

**Published:** 2021-08-31

**Authors:** Nicoleta Suciu, Cristina Oana Mărginean, Lorena Elena Meliț, Dana Valentina Ghiga, Cristiana Cojocaru, Cosmin O. Popa

**Affiliations:** 1 European and Research Projects Department, “George Emil Palade” University of Medicine, Pharmacy, Sciences and Technology, 540136 Târgu Mureș, Romania; nico.suciu03@gmail.com; 2 Department of Pediatrics I, “George Emil Palade” University of Medicine, Pharmacy, Sciences and Technology, 540136 Târgu Mureș, Romania; lory_chimista89@yahoo.com; 3 Department of Medical Scientific Research Methodology, “George Emil Palade” University of Medicine, Pharmacy, Sciences and Technology, 540136 Târgu Mureș, Romania; valentinaghiga@gmail.com; 4 Doctoral School of “George Emil Palade” University of Medicine, Pharmacy, Sciences and Technology, 540136 Târgu Mureș, Romania; cristiana-manuela.cojocaru@umfst.ro; 5 Department of Ethics and Social Sciences, “George Emil Palade” University of Medicine, Pharmacy, Sciences and Technology, 540136 Târgu Mureș, Romania; cosmin.popa@umfst.ro

**Keywords:** medical students, DECAS, BWAS, clinical communication skills

## Abstract

The aim of this study was to assess the level of creativity and personality traits and their mutual influence on medical students using the DECAS and BWAS. We performed a prospective descriptive study on 119 medical students from Târgu Mureș, Romania between November 2020 and July 2021, who answered questions relating to the BWAS and DECAS scales to evaluate their creativity and personality traits. Our findings pointed out a reverse correlation between age and both the original and revised BWAS (r = −0.2037, *p* = 0.0263; r = −0.1931, *p* = 0.0354). In terms of extraversion, we found a significant positive correlation for both openness (r = 0.3032, *p* = 0.0008) and emotional stability (r = 0.2868, *p* = 0.0016) and a negative correlation between extraversion and agreeability (r = −0.2394, *p* = 0.0087). Regarding creativity, we found positive correlations between emotional stability and both the original and revised BWAS (r = 0.20, *p* = 0.0279; r = 0.20, *p* = 0.0245). Medical students’ creativity might be positively influenced by emotional stability and seems to decrease with age. Higher extraversion scores could be related to increased openness and emotional stability as well as decreased agreeability.

## 1. Introduction

Personality is defined as the differences regarding how a person expresses feelings, emotions, thinking, and behaviors [1]. Different types of personalities might have either a positive or a negative impact on communication skills [2]. Communication is a very useful tool for medical students, especially in terms of doctor–patient communication influencing the patient’s trust in the physician’s medical judgment, his or her compliance to treatment, and eventually his or her chance to become healthy [3].

Communication skills are essential for each physician because they have to explain properly the diagnosis and enable the patients to understand the procedures and the treatment recommended for their conditions. Moreover, they have to build a solid partnership with both the patient and his or her family based on mutual respect inspiring trust in order to obtain the correct information regarding the patient’s medical history. Therefore, physicians must show empathy and compassion for their patients [4]. There are certain particular medical situations that require specific communication skills, such as the pediatrics field, where the doctor should be able to build a communication ”triangle” by gaining both the child’s and parents or caregivers’ trust [5]. Effective communication is the cornerstone of care, which certifies the response to the patient’s needs and family dynamic being centered on the patient’s and family’s care [5,6].

There are different scales used to assess the human personality. Thus, the “big five model” and its derivates such as the Personality Inventory (NEO PI-R) are the most commonly used tools for assessing personality [7]. The big five model is an instrument used to assess the five dimensions of personality: openness, extraversion, conscientiousness, agreeability, and emotional stability or neuroticism [8]. Each of these dimensions has six facets: openness (fantasy, aesthetics, feelings, actions, ideas, and values); extraversion (warmth, gregariousness, assertiveness, activity, excitement seeking, and positive emotions); conscientiousness (competence, order, dutifulness, achievement striving, self-discipline, and deliberation); agreeability (trust, straightforwardness, altruism, compliance, modesty, and tender-mindedness); and neuroticism (anxiety, angry hostility, depression, self-consciousness, impulsiveness, and vulnerability) [9]. Thus, the five-factor model, or the big five model, defines the underlying qualities of the different personality traits, and it has been assessed in multiple studies from different countries and cultures [10,11]. Nevertheless, this approach has been relatively ignored in terms of medical education, although it might provide an insight regarding the influence of medical students’ personality traits on their professional success [12].

The big five model was previously used to describe personality dimensions and their correlations to other aspects related to academic performance, burnout, and mental and physical health in medical students worldwide. A recent study performed on a large sample of Chinese medical students pointed out a relation between personality traits and resilience. Thus, the agreeableness, conscientiousness, and openness personality dimensions were associated with increased resilience to stressful situations and low levels of anxiety [13]. Another study involving medical students from the United Kingdom which assessed the relationship between the big five personality traits, perfectionism, and psychological distress concluded that low levels of conscientiousness and emotional stability were associated with poor mental health [14]. Moreover, the most common personality traits found in medical undergraduates from Egypt were agreeableness and openness [15]. Another field of study related various personality dimensions to the preference of a certain specialty among the medical students. For example, a recent exploration found that medical students in Jordan who displayed high extraversion, conscientiousness, and emotional stability preferred to choose a practical clinical specialty [16]. The association between personality traits, tolerance to uncertainty, and the specialty option was also explored on a sample of medical undergraduates from Buenos Aires, Argentina, suggesting that the students’ tolerance to ambiguity was higher among sixth year undergraduates than among freshmen [17].

Our investigation is also based on existing data regarding the relevance of communication skills, particularly the expression of empathetic concern, in medical contexts. According to Melchers et al., specific personality dimensions conceptualized according to the big five model correlate with empathy levels, especially for agreeableness and conscientiousness, which were predictors of cognitive and affective empathy across different cultures [18]. However, other research has shown that empathetic, active listening and assertiveness could also be predicted by the extraversion and openness personality dimensions [19]. Regarding the association between various personality traits and empathy, controversial results have been outlined in the literature. For instance, some recent evidence showed that empathy was only weakly correlated with agreeableness and revealed instead a strong negative association to low levels of emotional stability [20]. According to Popa et al., the personality is a strong predictor for academic success, especially in terms of a medical career [21]. Moreover, other authors emphasized that openness and conscientiousness along with a low level neuroticism are the key to good performance in the academic area [22,23]. Similarly, Hurtz et al. observed that employment and dynamism used in academic activities define and are correlated with openness and extraversion [24]. Based on these controversial results, it would be interesting to explore the correlation between communication skills and personality traits in our sample composed of medical students. Moreover, given that medical contexts display a wide spectrum of interindividual differences, it was hypothesized that clinical communication is strongly associated with creativity [25]. In addition, increasing challenges inherent to the medical field require that physicians act creatively in order to improve and streamline their clinical work [26].

Based on the aforementioned information, we hypothesize that students’ personality traits shape the doctor–patient partnership, influencing both academic success and the patient’s outcome.

Due to these statements, the aim of this study was to assess the level of creativity as well as the personality traits and their mutual influence on medical students using the DECAS and BWAS.

## 2. Materials and Methods

### 2.1. Study Design

We performed a correlational cross-sectional study on students of the Faculty of General and Dental Medicine from the “George Emil Palade” University of Medicine, Pharmacy, Sciences and Technology in Târgu Mureș between November 2020 and July 2021, who agreed to answer questions for the BWAS and DECAS in order to assess their creativity and personality traits.

### 2.2. Participants

We included in the study all the students of the Faculty of General Medicine and Dental Medicine enrolled at the “George Emil Palade” University of Medicine, Pharmacy, Sciences and Technology in Târgu Mureș independent of the year of study and age who agreed to participate. None of the included students benefited so far from any courses of communication with patients or other types of training focused on developing or improving personality attributes or creativity. Aside from the two scales, we also assessed the following demographic parameters: age, gender, year of study, and field of study (general medicine or dental medicine). We excluded from the study the students who refused to sign the informed consent as well as those who did not fill both questionnaires.

Before enrolling in the study, all participants completed and signed the informed consent regarding their inclusion in the study. After this initial phase, each student received via email two separate invitations for filling out the online questionnaires used in the research, and after providing their answers to each scale, they received electronic reports that were automatically generated by the software implemented for psychological testing and also via email. Each individualized report consisted of the participant’s particular scores at different personality dimensions that were explored, as well as their creativity levels.

### 2.3. Measures

The DECAS personality inventory is a modern psychometric tool developed by Sava A. et al. to evaluate the personality according to the five major personality factors [27]. The significance of each dimension of the DECAS depends on the scores obtained by the participants for each scale. Therefore, the openness personality dimension reveals aspects related to the individual’s culture, intellectual interests, and openness to experiences [28]. Extraversion indicates the sociability, energy, enthusiasm, task perseverance, and orientation toward others [27,29]. Conscientiousness is a dimension of the personality which generates the need for structure and order, prudence, responsibility, and perseverance in action or moral integrity. Agreeability refers to the impact of interpersonal relationships, consisting of trust in people, direct behavior, altruism, goodwill, modesty, and gentleness. Emotional stability (neuroticism) is related to the tendency to worry, depression, anxiety, impulsivity, and vulnerability [27]. The acronym DECAS comes from D: Deschidere (openness); E: Extraversiune (extraversion); C: Conștiinciozitate (conscientiousness); A: Agreabilitate (agreeability); and S: Stabilitate emoțională (emotional stability). The DECAS personality inventory encompasses two factors for preserving the psychometric properties of the instrument, namely the RD (random answers) and the AP (approval) validation scales, respectively. The RD validation scale is a factor sensitive to the subject’s tendency to give random answers, with a score higher than 70 T-levels leading to invalidation of the protocol. The AP validation scale is a factor sensitive to the subject’s tendency to respond more with “true” or, on the contrary, with “false”, and a score higher than 65 for the T-quotas or lower than 35 for the T-quotas leads to the invalidation of the protocol. [27].

The Cronbach’s alpha internal consistency coefficient was calculated based on each personality dimension for a sample of 1552 people. Thus, the Cronbach’s alpha internal consistency coefficient was 0.71 for openness, 0.75 for extraversion, 0.70 for conscientiousness, 0.71 for agreeability, 0.74 for emotional stability, 0.72 for social desirability, and 0.82 for approval [27]. Cronbach’s alpha for the DECAS was 0.677 (questionable), and for the BWAS, it was 0.962 (excellent).

The Barron Welsh Art Scale (BWAS) is a nonverbal instrument for the assessment of creativity. The scale was originally developed by Frank Barron based on the Welsh Figure Preference Test [30]. The BWAS consists of 86 items depicted as images, rated on a dichotomous scale with “Yes” or “No” as the response alternatives to the question of whether the subjects like a certain image or not, with higher scores representing elevated levels of creativity. If the respondents cannot decide if they like the image or not, they are asked to guess the answer. The main advantages of the instrument are that the administration does not require verbal knowledge or skills and the task does not involve attentional focus or advanced cognitive abilities for long periods of time. The instrument is composed of two scales. The first BW scale was elaborated based on comparisons of the answers offered by highly creative people for different items. However, several drawbacks of the initial scale were observed during factorial analysis, including the fact that item scoring was disproportionate. Therefore, the scale was revised, and an alternative scale named RA was developed, including an identical number of items rated as “Yes” and “No”. The scale was validated in various populations, including students, people active in artistic fields, or patients with psychiatric disorders or various disabilities [30]. The instrument provided excellent psychometric properties, with reliability coefficients over 0.95 for both BWAS subscales [31]. Moreover, according to Eysenck et al., psychoticism as a personality dimension is related to creativity and is evaluated very well by the BWAS [32]. In the current study, the BWAS was administered online via a specialized platform provided by the TestCentral company, which is a distributor of scientific psychological tests in Romania [33]. A report consisting of T-scores for both the original and the revised versions of the scale were generated for each subject immediately after completion.

### 2.4. Compliance with Ethical Standards

The study was designed and performed according to the principles of the American Psychological Association (APA) regarding ethical standards in psychological research. The research was approved by the Ethics Committee of the University of Medicine, Pharmacy, Sciences and Technology in Târgu Mureș (No. 1157/20.10.2020 and No. 1439/08.07.2021), and the participants signed their informed consent in order to be included in the investigation.

### 2.5. Statistical Analysis

The statistical analysis included elements of descriptive statistics (frequency, percentage, mean, median, standard deviation, and calculation of 95% confidence intervals), as well as elements of inferential statistics. The Shapiro–Wilk test was applied to determine the distribution of the analyzed data series. The Spearman and Pearson tests were applied to measure the magnitude of the correlation between the studied variables. We applied the Spearman correlation where the data series did not pass the normality test (without Gaussian distribution) and the Person correlation where both series had Gaussian distributions. For comparison of the means, we used the Student’s t-test for unpaired data and the Mann–Whitney test, a non-parametric, for comparison of the medians. The significance threshold chosen for the p-value was 0.05. The statistical analysis was performed using the GraphPad Prism software trial version (GraphPad Software, San Diego, CA, USA).

## 3. Results

Of the 380 participants who were invited to participate in the study, only 119 agreed to sign the informed consent and fill out the questionnaires. Thus, our final sample included 119 medical students, of whom 95 (79.83%) were females and 24 (20.17%) were males. The mean age of the whole group was 29.41 ± 4.090 years.

Our findings pointed out a reverse correlation between age and both BWAS versions (BWAS original scale r = −0.2037, *p* = 0.0263; BWAS revised scale r = −0.1931, *p* = 0.0354), indicating that older ages were significantly correlated with lower scores for both BWAS versions. We found no significant correlation between age and the individual parameters (i.e., openness, extraversion, conscientiousness, agreeability, and emotional stability). All the aforementioned correlations are detailed in Table 1.

Taking into account that medical students develop communication skills throughout their study years, we divided our sample into two subgroups: the first group involving students enrolled in the first three years (the preclinical years) and the second one formed by students from the last three years of study (clinical years). We compared the two scales between the two subgroups. Therefore, the only significant difference identified was for extraversion (*p* = 0.0004), suggesting that students from their preclinical years express a higher level of extraversion when compared with those enrolled in their clinical years. The comparison of the two sales is detailed in Table 2.

In terms of extraversion, we found a significant positive correlation with both openness (r = 0.3032, *p* = 0.0008) and emotional stability (r = 0.2868, *p* = 0.0016), pointing out that higher extraversion scores were related to increased openness and emotional stability. Contrary to that, we encountered a negative significant correlation between extraversion and agreeability (r = −0.2394, *p* = 0.0087), indicating that increased extraversion was associated with lower agreeability scores. In addition, our study revealed a positive correlation between emotional stability and creativity, since we found significant positive correlations between emotional stability and both the BWAS original scale (r = 0.20, *p* = 0.0279) and BWAS revised scale (r = 0.20, *p* = 0.0245). All the assessed parameters are described in Table 3.

## 4. Discussions

Our study aimed to assess certain essential personality traits in a group of medical students from Romania. Thus, our findings revealed a significant positive correlation between age and the BWAS, indicating that older ages were significantly correlated with lower creativity scores. Moreover, we found that medical students’ extraversion was positively correlated with both openness and emotional stability but not with agreeability, which was proven to be negatively influenced by extraversion. In terms of emotional stability, we showed that medical students who displayed an optimal level of emotional stability expressed a higher degree of creativity. Regarding the level of education, we noticed that students in their preclinical years (first three years of study) displayed a higher level of extraversion when compared with those in their clinical years (last three years of study). This finding is rather confusing, since it is generally assumed that personality traits develop throughout the study years as the student grows, and it clearly indicates that the implementation of proper training on this topic in medical students is crucial.

The medical profession is by far the most challenging of all, since its success depends not only on the physician’s cognitive abilities and medical skills but also on his or her personality, which displays a great influence on the doctor–patient relationship, also known as the key to a patient’s best outcome. In order to build a strong relationship with his or her patients, the doctor’s behavior must include empathy, collaboration, responsibility, respect, positive thinking, inoculation of hope, and also identifying physical, cultural, psychological, and social barriers that hinder effective communication [5]. It is a well-known fact that both verbal and nonverbal communication present equal importance in addressing the patient’s and family’s cognitive and affective needs [32]. The techniques which facilitate communication are empathy, reflection, clarification, and mirroring [32]. The innate traits of a personality are decisive for establishing a close and solid doctor–patient relationship, which will definitely improve the patient’s outcome, augmenting the therapeutic effect of the medical treatment. Nevertheless, all these abilities can also be taught and improved by implementing proper clinical communication skill training in medical students.

Academic performance is definitely influenced by personality traits independent of medical or non-medical settings [34]. The genetic background has a strong influence (up to 80%) on personality traits, which subsequently impact long-term behavior [35], a fact that might explain the lack of correlation between age and the DECAS in our study. While it is true that personality traits might be taught with proper training, they depend mostly on the individual’s innate personality, thus being unlikely to differ with age. In terms of medical professions, these traits were associated with mental health, career success, devotement toward the profession, the learning approach, as well as academic performance [36,37,38,39,40]. It is worth mentioning that medical educators should be aware of the impact of personality traits on a student’s performance, since they display a strong influence in the setting of extreme stress [41,42,43]. Thus, the five-factor model should be used as a theoretical basis by medical educators in order to achieve the best outcome in a medical student’s education. Moreover, a study that assessed this model in first-year medical students from Malaysia proved that extraversion, conscientiousness, agreeability, and openness had a positive impact on the stress, depression, and anxiety of these students in stressful conditions, whereas a negative influence was underlined for neuroticism [34]. These findings might be extrapolated to the medical profession, suggesting that the above-mentioned personality traits guide the medical performance. Other meta-analyses emphasized that extraversion might be defined as the mirror of wellbeing, since it is closely related to life quality and satisfaction, happiness, and positive, negative, and overall effects [44,45]. In spite of the moderate degree of correlation, our findings also revealed a significant correlation between extraversion and both emotional stability and openness (r = 0.2868, *p* = 0.0016; r = 0.3032, *p* = 0.0008). Nevertheless, our study underlined that increased extraversion might be associated with lower agreeability scores. Taking into account that McCrae and Costa showed that agreeability is associated with altruism, trust, sympathy, and cooperation [46], we can state, based on our findings, that these attributes might be negatively influenced by extraversion. Nevertheless, further studies are needed with larger samples, since our statistical analysis proved only a moderate degree of correlation between the aforementioned parameters (r = −0.2394, *p* = 0.0087).

Personality traits are also essential in guiding the medical student to choose the most suitable specialty, with subsequent major implications for the development of healthcare systems [47]. Thus, medical educators should take into account and increase their knowledge regarding a student’s personality traits in order to provide them appropriate career counseling. A recent study performed on this topic proved that students with increased levels of openness preferred medical departments, while those with an overexpression of agreeability tended to choose clinical medicine [47]. Moreover, the same study pointed out that students with increased levels of openness reported satisfaction and personal interest as the most important factors in choosing their specialty, while those with higher levels of conscientiousness stated that personal interest mostly guided their choice [47]. In terms of age, the authors underlined that older students tended to be more conscientious and agreeable [47]. On the contrary, in our study, we found no significant correlation between age and any of the five parameters included in the DECAS inventory. Furthermore, we noticed that students in their preclinical years expressed a higher level of extraversion compared with the older ones, who were enrolled in the last three years of study (i.e., the clinical years).

Interprofessional cooperation between different health care providers is a highly desirable skill for well-functioning teams, which in the medical profession conveys the patient’s safety and improves his or her outcome [48]. However, interprofessional collaboration might be challenging and troublesome in certain personalities if not trained properly. Therefore, interprofessional education was meant to develop the student’s collaboration skills in order to achieve better interprofessional teamwork, with its implementation being promoted by the World Health Organization [49,50]. The student’s attitude toward interprofessional education and interprofessional collaboration depends on multiple intrinsic and extrinsic aspects. Among the intrinsic factors, gender is an unmodifiable factor that was proven to influence this attitude, since female students showed a more positive attitude toward this type of collaboration and teamwork [51,52,53]. Taking into account that almost 80% of our respondents were females, we might emphasize that females expressed higher levels of positive attitudes and openness to challenges. Personality traits represent another intrinsic dimension that influence medical choices and interprofessional collaboration, but fortunately, with proper education, the unfavorable traits might be diminished in order to improve these skills.

Medical empathy is a hallmark for the medical profession, and it defines the ability to understand a patient’s concerns, experiences, and perspectives, as well as to express this understanding in order to help him or her [54]. A recent study underlined that empathy is related to the personality, suggesting that personalized intervention strategies could increase empathy in medical students [55]. A thorough study and understanding of relationships between empathy and personality traits is essential for predicting possible behaviors, guiding specialty choice in medical students, or even for the selection of future students that match the medical school [55]. Several studies that focused on assessing the impact of the personality on empathy encountered positive associations between empathy and both agreeability and openness [56,57,58]. Aside from openness, Guilera et al. showed that the empathy quotient was also positively associated with conscientiousness and extraversion [56]. It is worth mentioning that affective and cognitive empathy are two different aspects and that certain dimensions of empathy might be modulated and improved by personalized intervention strategies [55]. Thus, two recent studies focused on improving cognitive empathy by promoting behaviors related to agreeability, such as patient-centered attention [59,60]. The authors indicated that affective empathy can also be improved by controlling neuroticism and anxiety, especially in medical students with people-oriented specialty preferences [59,60]. Therefore, the proper understanding of personality traits in medical students will help educators guide them to better choose a suitable specialty which will definitely result in a major improvement of the patient’s outcome and a considerable decrease in the burden on healthcare systems.

As we already mentioned above, the BWAS is a tool designed for assessing creativity, and it was previously used especially in patients with mental conditions such as schizophrenia or bipolar disorder [61,62]. Moreover, this scale proved to also be useful in comparing the creativity between mood disorder patients, healthy controls, and highly creative individuals [63]. Nevertheless, to the best of our knowledge, this is among the few studies, if not the first, which assessed the level of creativity in medical students using the BWAS. Our findings outlined that creativity decreased with age, and it might have been positively influenced by emotional stability. Nevertheless, we identified only a mild degree of correlation between the parameters (BWAS original scale r = 0.20, *p* = 0.0279; BWAS revised scale r = 0.20, *p* = 0.0245), requiring further studies on this topic. Similar findings were reported by Strong et al., who stated that neuroticism and openness could enable affective and cognitive advantages to increase creativity [64].

Finally, the main purpose of our study was to obtain an overall picture regarding the representation of specific personality traits among medical students in order to develop and implement targeted and comprehensive communication training courses. Our statement is based on recent evidence indicating that sustained educational interventions with repetitive reinforcements to enhance empathy are the most suitable for medical students [65]. Along this line, we could consider the idea of developing and implementing long-lasting programs for improving communication skills in medical students throughout their entire academic process.

The limitations of this study are a relatively small sample of subjects, the fact that they were enrolled from a single medical university from Romania, and that we could not assess the relationship between their personality traits and the medical communication experienced in clinical settings. It is also important to mention that we cannot rule out the fact that students might have improved their responses to meet our expectations. Thus, further studies would be extremely useful with larger samples from multiple medical universities in Romania to assess the influence of personality on practical communication skills in students. Moreover, a comparison of these skills before and after proper clinical communication education would definitely underline the essential role of communication training in medical universities.

## 5. Conclusions

Personality traits express a major influence on medical students’ clinical skills and their future professional success. Among these, extraversion is probably one of the most important attributes since it defines wellbeing, and according to our study, it positively influences openness and emotional stability. Nevertheless, an increased level of extraversion could be associated with lower agreeability. We also underlined that optimal emotional stability improved creativity among medical students. Our findings sustain the critical need for implementing proper training and education among medical students in terms of communication, empathy, and even certain personality traits, which will enable them to further develop their clinical skills.

## Figures and Tables

**Table 1:** The Spearman correlation between the DECAS personality inventory, BWAS, and age.

Parameters	Age		
	r Coefficient	95% Confidence Interval	*p* Value *
D (Openness)	−0.07214	−0.2540 to 0.1146	0.4356
E (Extraversion)	−0.1759	−0.3497 to 0.009691	0.0557
C (Conscientiousness)	0.07415	−0.1126 to 0.2559	0.4229
A (Agreeability)	−0.05612	−0.2389 to 0.1305	0.5444
S (Emotional Stability)	0.06351	−0.1232 to 0.2459	0.4926
BWAS (original)	−0.2037 *	−0.3748 to −0.01919	0.0263
BWAS (revised)	−0.1931 *	−0.3653 to −0.008127	0.0354

Legend: Barron Welsh Art Scale (BWAS), DECAS (D: Deschidere (openness); E: Extraversiune (extraversion); C: Conștiinciozitate (conscientiousness); A: Agreabilitate (agreeability); and S: Stabilitate emoțională (emotional stability)). * Spearman correlation was used at *p* < 0.05 (two-tailed).

**Table 2:** Comparison of BWAS and DECAS between preclinical and clinical years.

Parameters	Preclinical Years (*n* = 60) Means ± SD (Median Values)	Clinical Years (*n* = 59) Means ± SD (Median Values)	*p* Value *
D (Openness)	55.33 ± 10.30 (56.70)	53.89 ± 10.08 (54.40)	0.4423
E (Extraversion)	47.94 ± 12.48 (45.40)	41.63 ± 11.17 (42.60)	0.0004
C (Conscientiousness)	52.51 ± 11.17 (50.85)	52.98 ± 10.52 (52.00)	0.8132
A (Agreeability)	51.17 ± 7.669(51.20)	50.99 ± 8.801 (50.50)	0.9019
S (Emotional Stability)	45.09 ± 9.596 (43.20)	46.31 ± 9.188 (45.30)	0.4802
BWAS (original)	59.10 ± 14.62 (59.00)	55.08 ± 15.54 (55.00)	0.2073 *
BWAS (revised)	61.20 ± 15.50 (60.50)	56.59 ± 16.41 (56.00)	0.1289 *

Legend: Barron Welsh Art Scale (BWAS), DECAS (D: Deschidere (openness); E: Extraversiune (extraversion); C: Conștiinciozitate (conscientiousness); A: Agreabilitate (agreeability); and S: Stabilitate emoțională (emotional stability)]. * Mann–Whitney test.

**Table 3:** The Pearson correlation between different personality types.

	D			E			C			A			S		
	r	95% CI	*p*	r	95% CI	*p*	r	95% CI	*p*	r	95% CI	*p*	r	95% CI	*p*
D	1			0.3032 **	0.1303 to 0.4582	0.0008	0.1691	−0.0166 to 0.3436	0.0660	−0.1365	−0.3089 to 0.0447	0.1389	0.0098	−0.1705 to 0.1895	0.9158
E	0.3032	0.1303 to 0.4582	0.0008	1			−0.0831	−0.2643 to 0.1037	0.3690	−0.2394 **	−0.4021 to −0.0621	0.0087	0.2868 **	0.1126 to 0.4439	0.0016
C	0.1691	−0.0166 to 0.3436	0.0660	−0.0831	−0.2643 to 0.1037	0.3690	1			−0.1037	−0.2835 to 0.0832	0.2618	−0.0327	−0.2166 to 0.1535	0.7244
A	−0.1365	−0.3089 to 0.0447	0.1389	−0.2394 **	−0.4021 to −0.0621	0.0087	−0.1037	−0.2835 to 0.0832	0.2618	1			0.0282	−0.1526 to 0.2072	0.7607
S	0.0098	−0.1705 to 0.1895	0.9158	0.2868 **	0.1126 to 0.4439	0.0016	−0.0327	−0.2166 to 0.1535	0.7244	0.02821	−0.1526 to 0.2072	0.7607	1		
BWAS (original)	0.1390	−0.0474 to 0.3161	0.1316	0.0363	−0.1500 to 0.2200	0.6953	−0.1419	−0.3188 to 0.0445	0.1236	0.04230	−0.1441 to 0.2258	0.6479	0.2016 *	0.0169 to 0.3729	0.0279
BWAS (revised)	0.1611	−0.0248 to 0.3363	0.0800	0.0272	−0.1589 to 0.2113	0.7694	−0.1305	−0.3083 to 0.0561	0.1571	0.04816	−0.1383 to 0.2313	0.6030	0.2062 *	0.0218 to 0.3770	0.0245

Legend: Barron Welsch Art Scale (BWAS), DECAS (D: Deschidere (openness); E: Extraversiune (extraversion); C: Conștiinciozitate (conscientiousness); A: Agreabilitate (agreeability); and S: Stabilitate emoțională (emotional stability)]. * *p* < 0.05 (two-tailed). ** *p* < 0.01 (two-tailed).

## Data Availability

Not applicable.
